# 

**Published:** 1920-11-06

**Authors:** 


### THE HOSPITAL

**The Modern Newspaper of Administrative Medicine and Institutional Life.**

Telegraphic Address: OSPEDALE, RAND, LONDON. Telephone Number: 2734 GERRARD.

No. 1796, Vol. LXIX. | Saturday, November 6th, 1920. | PRICE TWOPENCE.

